# Robot-assisted vs traditional percutaneous freehand for the scaphoid fracture treatment: a retrospective study

**DOI:** 10.1007/s00264-022-05532-9

**Published:** 2022-08-04

**Authors:** Chengwei Xiao, Dan Wei, Zongdong Zhu, Hui Chen, Weijun Zhou, Xiaoming Tang, Jiabin Yuan, Yue Wang, Jiang Hu

**Affiliations:** 1 Orthopaedic Department, Sichuan Provincial People’s Hospital, University of Electronic Science and Technology of China, 32# W. Sec 2, 1st Ring Rd, Qingyang District, Chengdu, 610072 China; 2 Chinese Academy of Sciences Sichuan Translational Medicine Research Hospital, Chengdu, 610072 China

**Keywords:** Scaphoid fracture, Cannulated screw fixation, TiRobot, Traditional percutaneous freehand

## Abstract

**Purpose:**

The purpose of this study was to assess the efficiency, safety, and accuracy of cannulated screw fixation using a robot-assisted method compared with a traditional percutaneous freehand method.

**Methods:**

This retrospective clinical study included 18 patients with scaphoid fracture who underwent cannulated screw fixation by robot-assisted technique or traditional percutaneous freehand technique from June 2018 to June 2020. All patients were divided into the robot-assisted group (9 patients) or the traditional surgery group (9 patients). The operation time, blood loss, number of intra-operative fluoroscopies, fracture healing time, Mayo wrist function score, and screw implantation accuracy were recorded in the two groups.

**Results:**

The average age of the robot-assisted group was 37.9 ± 10.6 years (with a range of 30 to 52 years), there were eight males and one female, and there were six cases of scaphoid fracture on the right side and three on the left side. The average pre-operative time was 2.8 ± 0.7 days (ranging from 1 to 3 days). The average age of the traditional surgery group was 31.6 ± 6.8 years (with a range of 20 to 45 years), there were eight males and one female, and there were five cases of scaphoid fracture on the right side and four on the left side. The average pre-operative time was 2.1 ± 0.8 days (with a range of 2 to 4 days). The number of intra-operative fluoroscopies was 24.4 ± 3.5 in the traditional surgery group, whereas it was only 10.1 ± 1.9 in the robot-assisted group, which was significantly lower (*P* < 0.05). The average operation time of the traditional operation group was 48.4 ± 12.2 min, and that of the robot-assisted group was 32.6 ± 4.2 minutes, which was significantly shorter (*P* < 0.05). The angles between the actual screw position and the central axis of the scaphoid on both the coronal and sagittal post-operative CT images were 8.3° ± 2.3° and 8.8° ± 1.6° for the traditional operation group and 3.8° ± 0.8° and 4.3° ± 1.2° for the robot-assisted group, so the accuracy of the robot-assisted group was significantly higher (*P* < 0.05). There were no significant differences between the two groups in wrist function recovery or fracture healing time.

**Conclusion:**

Robot-assisted treatment of scaphoid fracture is more accurate than traditional freehand technology, with shorter operation time and fewer intra-operative fluoroscopies. There is no difference between the two surgical techniques in intra-operative bleeding, post-operative fracture healing, or functional recovery. Robot-assisted surgery is a safe, effective, and accurate method for treating scaphoid fracture.

## Introduction

Scaphoid fractures account for 50–80% of carpal fractures and 3% of wrist fractures. The annual incidence is 29/100,000, most often occurring in young adults [1, 2]. The healing of scaphoid fracture has always been a difficult problem in clinical treatment because of the scaphoid’s special anatomy and scarce blood supply. When scaphoid fracture is treated with plaster fixation, healing usually takes eight to 12 weeks, but compression cannulated screws can speed up the healing rate. Accurately placing screws is the key to this surgical treatment, which requires repeated fluoroscopy to confirm. Robot-assisted technology developed in recent years offers accurate screw placement and has been applied in orthopaedics [3]. For example, minimally invasive pedicular screw grafting has been widely used to treat vertebral fracture [4, 5]. Robot-assisted techniques have also been effectively used in the field of trauma orthopaedics for the post-operative rehabilitation of femoral neck fracture, pelvic fracture, and distal radius fracture [6–8]. Moreover, the use of robot-assisted technology in scaphoid fracture and nonunion has achieved good clinical results [3, 9]. This study retrospectively analyzed the operation time, blood loss, number of intra-operative fluoroscopies, fracture healing time, Mayo wrist function score, and screw implantation accuracy of robot-assisted fixation and traditional percutaneous freehand fixation in the treatment of non-displaced scaphoid fractures.

## Materials and methods

From June 2018 to June 2020, 18 cases of new non-displaced scaphoid fractures received surgical treatment in Sichuan Provincial People’s Hospital, University of Electronic Science and Technology of China, including nine cases of traditional freehand screw placement and nine cases of robot-assisted screw placement. All procedures were performed by one experienced surgeon. The demographic data, injury side, and the pre-operative time were collected. Additionally, the operation time, blood loss, number of intra-operative fluoroscopies (each fluoroscopy took the same amount of time), fracture healing time, Mayo wrist function score, and screw placement accuracy were recorded.

### Evaluation of screw implantation accuracy

**Fig. 1:**
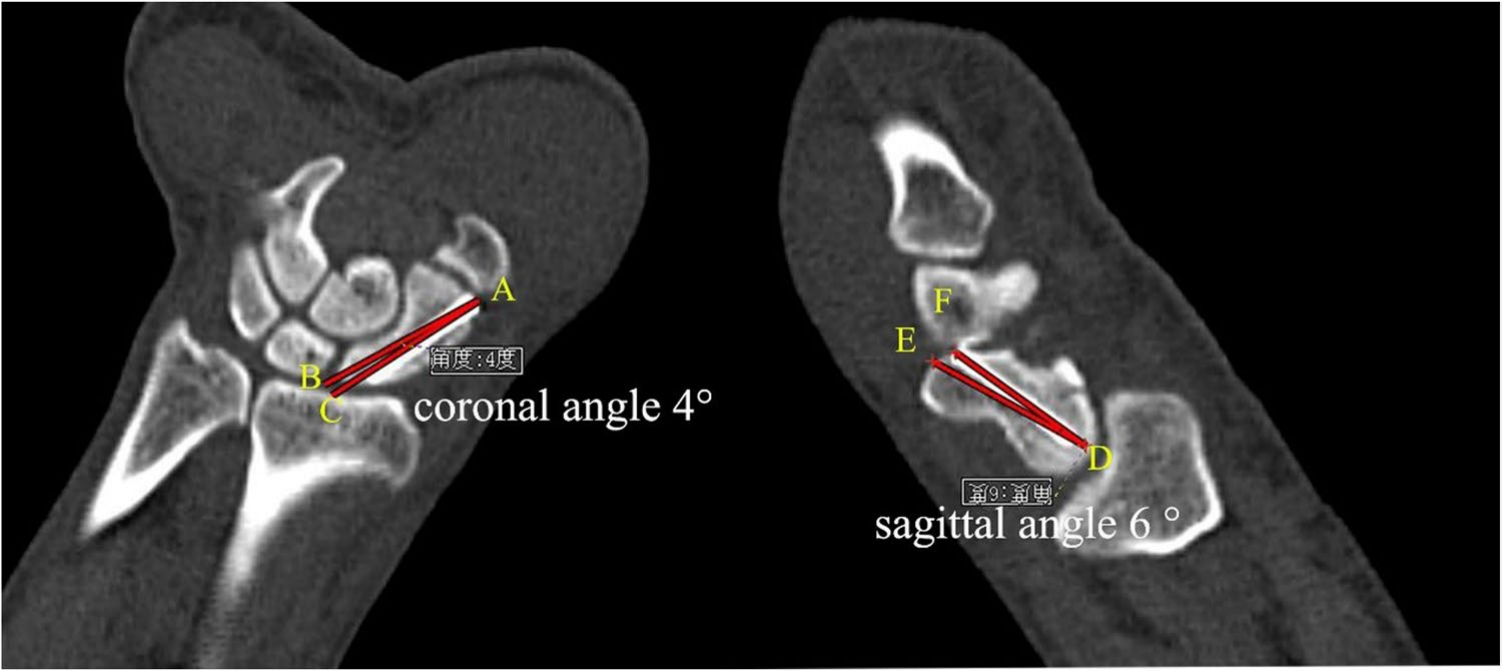
The screw implantation accuracy was evaluated by the angles between the actual position of the screw and the ideal position of the screw (i.e., the central axis of the scaphoid) in post-operative sagittal and coronal CT images

The screw implantation accuracy was evaluated by the angles between the actual position of the screw and the ideal position of the screw (i.e., the central axis of the scaphoid) in post-operative sagittal and coronal CT images (Fig. 1), which was the method used by Hoffmann et al. [10].

### Healing time and wrist function

The healing time of scaphoid fracture was determined through regular post-operative follow-up. The functional recovery of patients was assessed by Mayo wrist function score in the last follow-up.

### Operation techniques

#### Traditional percutaneous cannulated screw implantation

The patient was placed under brachial plexus block anesthesia. Then, the scaphoid tubercle in the neutral position of the wrist was found, and the entry point was located in the scaphoid tubercle via C-arm fluoroscopy. The forearm was rotated back to achieve dorsal extension of the wrist joint, and the scaphoid tubercle was kept toward the Lister tubercle line at an angle of 45° to the forearm, which can pass through the long axis of the scaphoid. The full length of the scaphoid was observed via scaphoid position fluoroscopy. The lateral position ensured that the Kirschner wire was located in the capitate and lunate space. At this time, the Kirschner wire was passed through the long axis of the scaphoid to the greatest extent, and the distal end of the guide wire was located behind the edge of the proximal pole of the scaphoid in the lateral fluoroscopy. After measuring the length, 4 mm was subtracted, and the far and near ends of the countersunk head of the cannulated screw were each 2 mm. Then holes were drilled with a hollow drill, and cannulated screws of appropriate length were driven into the holes.

#### Robot-assisted percutaneous cannulated screw implantation

**Fig. 2:**
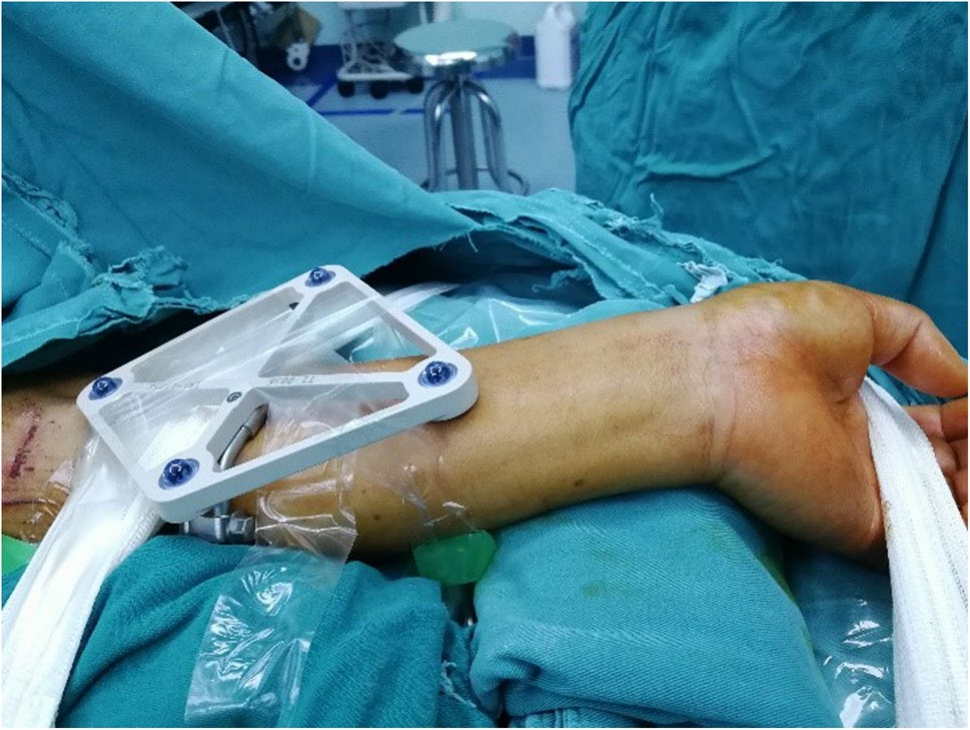
A sterile elastic bandage was used to fix the forearm and palm of the affected limb to avoid displacement during operation

The patient was placed under general anesthesia or brachial plexus anesthesia, and the affected limb was abducted 90°. After sterilization, a roll-shaped pad was shaped on the dorsal side of the wrist joint to make the wrist joint extend 20–25°, and the wrist was fixed on the abduction operation table with slight ulnar deviation. Additionally, a sterile elastic bandage was used to fix the forearm and palm of the affected limb to avoid displacement during operation (Fig. 2).

**Fig. 3:**
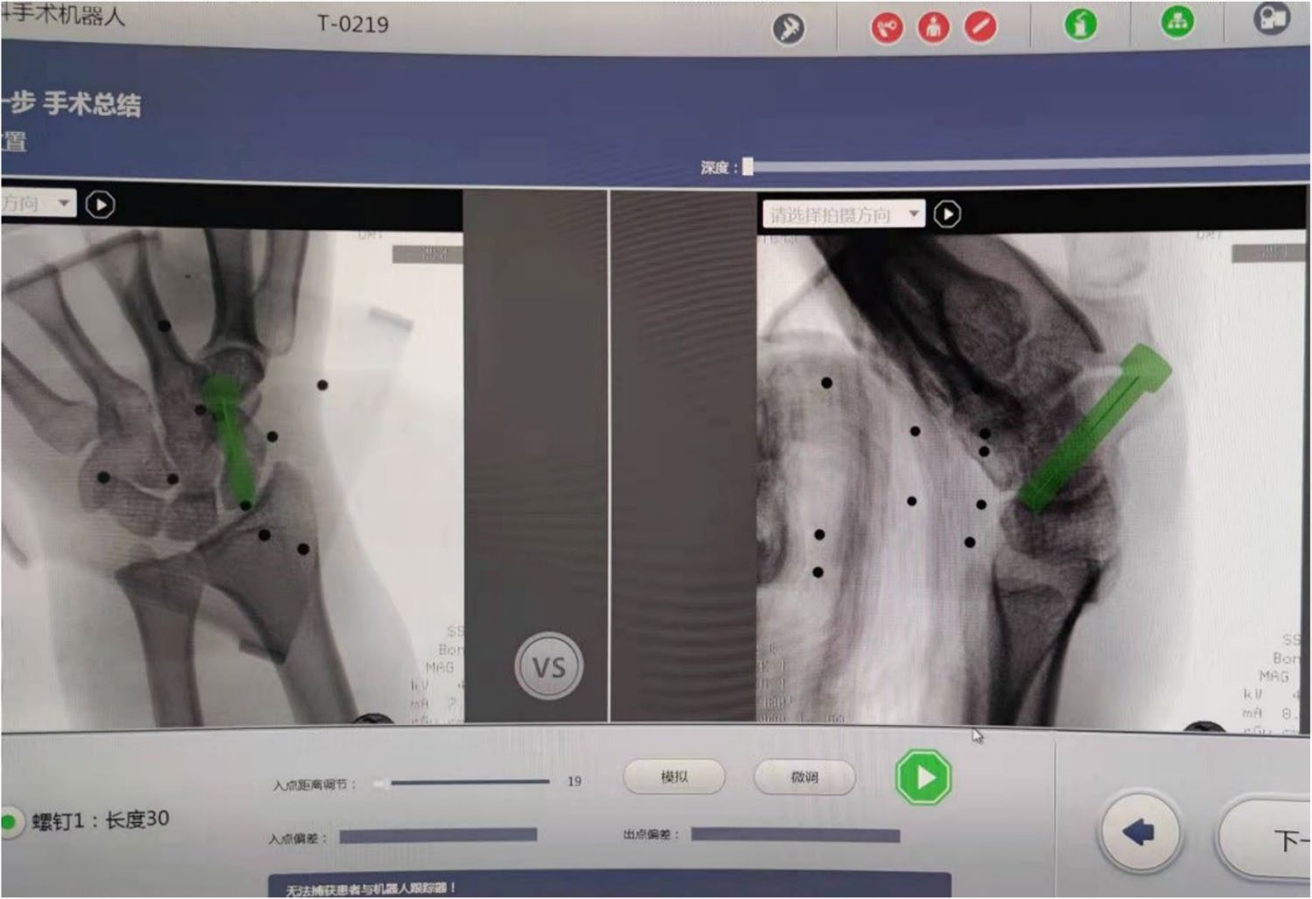
The operation platform displayed the planned screw insertion point and screw length

**Fig. 4:**
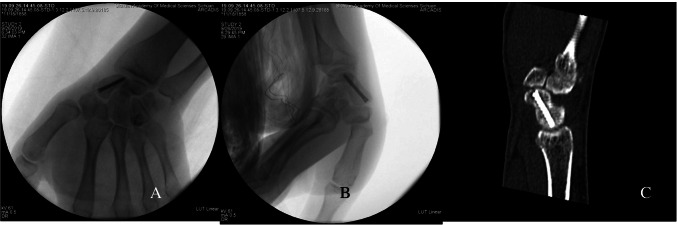
After the position of the screw was confirmed to be satisfactory, the cannulated screw was placed

The TiRobot TM navigation robot used in this study (Beijing Tianzhihang Medical Technology Co., Ltd., China) was composed of a multi-degree-of-freedom manipulator, an optical tracking device, a surgical planning and control platform, and surgical instruments that assist the robot to establish spatial coordinates. After sterilization and paving sterile sheet, a tracer was fixed on the skin of the forearm. The tracer can track the spatial change of the surgical guide wire in real time with an error of less than 0.3 mm. After the installation of instruments and devices, fluoroscopy was performed, including the anteroposterior and lateral position of the wrist joint. The screw position was planned by the surgeon on the operation platform according to the captured image data. After the planning was completed, the operation platform displayed the planned screw insertion point and screw length (Fig. 3). The manipulator moved to the corresponding position according to the planned path to provide a guide wire entry channel for screw placement. The surgeon implanted the guide wire along the channel, and then anteroposterior and lateral wrist joint fluoroscopy was performed with the C-arm machine. After the position of the screw was confirmed to be satisfactory, the cannulated screw was placed (Fig. 4).

### Statistical analysis

Statistical analysis was performed using SPSS version 26.0 (IBM, Corporation, Armonk, NY). Continuous variables were expressed as mean ± standard deviation (SD), and differences between continuous variables were assessed via the Student’s *t*-test. Differences between categorical variables were assessed via the chi-square test or Fisher’s exact test. A statistically significant difference was defined as *P* < 0.05.

## Results

### Patient characteristics

Patient characteristics are provided in Table 1. The average age of the nine patients in the robot-assisted (RA) group was 37.9 ± 10.6 years (with a range of 30 to 52 years). The RA group included eight males and one female, with six cases of scaphoid fracture on the right side and three on the left side. The average preoperative time was 2.8 ± 0.7 days (ranging from 1 to 3 days). The average age of the nine patients in the traditional percutaneous freehand (TPF) group was 31.6 ± 6.8 years (with a range of 20 to 45 years). The TPF group included eight males and one female, with cases of scaphoid fracture on the right side and four on the left side. The average preoperative time was 2.1 ± 0.8 days (with a range of 2 to 4 days). There were no significant differences in gender, age, injury side, or pre-operative time between the two groups (*P* > 0.05).

**Table 1:** Patient characteristics

Group	Population	Gender (M/F)	Injury side (R/L)	Pre-OP time (days)	Age
TPF	9	8/1	5/4	2.1 ± 0.8	31.6 ± 6.8
RA	9	8/1	6/3	2.8 ± 0.7	37.9 ± 10.6
*P*-value		1.0	1.0	0.069	0.151

The number of intra-operative fluoroscopies was 24.4 ± 3.5 in the TPF group and 10.1 ± 1.9 in the RA group (Table 2); the number of intra-operative fluoroscopies in the RA group was significantly less than that in the TPF group (*P* < 0.05). Regarding the operation time, the average time of the TPF group was 48.4 ± 12.2 min, and the average time of the RA group was 32.6 ± 4.2 min; the operation time of the RA group was significantly shorter than that of the TPF group (*P* < 0.05). The statistics of intra-operative blood loss showed that there was no significant difference between the TPF group and the RA group. The post-operative Mayo wrist function score of the TPF group was 95.6 ± 3.9, and that of the RA group was 97.7 ± 2.6; there was no significant difference in post-operative functional recovery between the two groups. The angles between the actual position of the screw and the position of the central axis of the scaphoid in both the coronal and sagittal CT images were 8.3° ± 2.3° and 8.8° ± 1.6° for the TPF group and 3.8° ± 0.8° and 4.3° ± 1.2° for the RA group; hence, screw placement was significantly more accurate in the RA group (*P* < 0.05). The post-operative fracture healing time was 11.1 ± 2.3 weeks in the TPF group and 10.2 ± 1.6 weeks in the RA group, so there was no significant difference.

**Table 2:** The number of intra-operative fluoroscopies

	Traditional group	Robot-assisted group	*P*-value
Radiation time	24.4 ± 3.5	10.1 ± 1.9	*P* = 0.001
Operation time (min)	48.4 ± 12.2	32.6 ± 4.2	*P* = 0.002
Blood loss (ml)	6.7 ± 2.5	6.1 ± 2.2	*P* = 0.624
Mayo wrist	95.6 ± 3.9	97.7 ± 2.6	*P* = 0.176
Angle deviation coronar	8.3 ± 2.3°	3.8 ± 0.8°	*P* = 0.002
Angle deviation sagittal	8.8 ± 1.6°	4.3 ± 1.2°	*P* = 0.001
Fracture union time (week)	11.1 ± 2.3	10.2 ± 1.6	*P* = 0.346

## Discussion

Percutaneous cannulated screw fixation of scaphoid fracture is a safe and effective surgical technique, although there are some potential surgical complications, such as tendon injury and median nerve injury [11, 12]. These complications can be effectively avoided through appropriate soft tissue exposure and intra-operative fluoroscopy. Implanting the screw into the center of the scaphoid by percutaneous fixation technology is a goal that has been long pursued by surgeons. To achieve this goal, multiple fluoroscopies need to be performed during operation with increasing radiation exposure.

Percutaneous cannulated screw fixation technology is divided into the volar approach and the dorsal approach. Jeon et al. confirmed that in percutaneous cannulated screw fixation of scaphoid fracture, the dorsal approach is more parallel to the central axis of the scaphoid and has a better vertical angle to the fracture line compared to the volar approach, but there is no significant difference between the two approaches in fracture healing time and post-operative function [13]. Lee et al. found that for percutaneous screw fixation of scaphoid fracture, different guide wire entry points should be selected according to the fracture site before the screw can be implanted in the center [14]. Using ultrasound guidance and 3D printed assistance technology to treat scaphoid fracture can effectively reduce intra-operative radiation [15–17]. The use of robot-assisted technology in the surgical treatment of traumatic fractures can provide more accurate reduction, and also enable less invasive procedures and less intra-operative radiation [6, 18]. Our study confirmed that the number of intra-operative fluoroscopies was significantly reduced when performing robot-assisted surgery instead of traditional percutaneous surgery for cannulated screw fixation.

### Advantages of robot-assisted technique

Tianji orthopaedic robot-assisted technology has been effectively applied in treating spine and traumatic fractures. Because the tracer in this technology can be used to track spatial changes of the surgical site in real time, and the error is less than 0.3 mm [19], the accuracy of screw implantation compared to the planned screw position is very high. In our study, the angle between the screw position and the central axis of the scaphoid was smaller when robot-assisted technology was used for screw fixation. Liu et al. used a special fixator for robot-assisted fixation of scaphoid fracture [3]. We improved their methodology by fixing the forearm on the operating table and fixing the tracer on the forearm skin to avoid new wounds caused by the tracer. A rolled-up drape was placed under the wrist to extend it to 60°. In addition, we used elastic bandages to fix the forearm and palm on the table to ensure that the scaphoid and wrist joints did not move. With this fixation method, there is no need for special wrist fixation equipment, and the wrist joint can be fixed in the ulnar deviation of extension, which enables effective operation.

Our study showed that the operation time and number of intra-operative fluoroscopies in the RA group were less than those in the TPF group. For traditional percutaneous freehand technique, the surgeon needs to adjust the position of the guide wire under fluoroscopy, so as to find the appropriate position for cannulated screw implant. The major time of operation was spent on adjusting the guide wire. The robot-assisted technique can accurately implant the guide wire into the ideal position only once, so it can effectively shorten the operation time. For radiation exposure, the traditional percutaneous freehand technique required fluoroscopy to determine the position after each change of the guide wire. Based on the accuracy of the Tianjin orthopaedic robot, it avoided the number of times it takes to adjust the guide wire. It can significantly reduce the times of fluoroscopy through an accurate implantation of the guide wire. Therefore, patients and surgical medical staff receive less radiation exposure during operation.

In traditional percutaneous freehand technique, the surgeon needs to fully understand the complex anatomical structure of the scaphoid, learn autopsy, and accumulate clinical experience in order to better master the percutaneous cannulated screw fixation technique. However, with the robot-assisted technique, the learning curve is significantly shortened. The surgeon only needs to master the conventional operation of the robot and accurately drive the guide wire and implant the screw with the help of the robot. Therefore, junior doctors can learn and master the technique in a short time.

### Limitations

Because the TiRobot system is relatively new and there is a low incidence of scaphoid fracture, only a small sample size was reported in our study. Additionally, this study was a retrospective study, and the follow-up period was short. In the future, a multicenter prospective study with a large number of cases and a long follow-up period should be performed, and guidelines for robot-assisted surgery should be made.

## Conclusion

In conclusion, robot-assisted screw fixation for treating scaphoid fracture is more accurate than traditional freehand screw fixation, with a shorter operation time and less intra-operative radiation. There is no difference between the two techniques in terms of fracture healing time or post-operative function outcome. In the future, a study with a larger sample size should be conducted. Robot-assisted treatment of scaphoid fracture is a safe, effective, and accurate surgical method.

## Data Availability

The datasets generated during or analyzed during the current study are available from the corresponding author on reasonable request.
